# Evolutionary Analysis of Dengue Serotype 2 Viruses Using Phylogenetic and Bayesian Methods from New Delhi, India

**DOI:** 10.1371/journal.pntd.0004511

**Published:** 2016-03-15

**Authors:** Nazia Afreen, Irshad H. Naqvi, Shobha Broor, Anwar Ahmed, Syed Naqui Kazim, Ravins Dohare, Manoj Kumar, Shama Parveen

**Affiliations:** 1 Centre for Interdisciplinary Research in Basic Sciences, Jamia Millia Islamia, New Delhi, India; 2 Dr. M.A. Ansari Health Centre, Jamia Millia Islamia, New Delhi, India; 3 Department of Microbiology, All India Institute of Medical Sciences, New Delhi, India; 4 Protein Research Chair, Department of Biochemistry, College of Science, King Saud University, Riyadh, Saudi Arabia; 5 Centre for Culture, Media & Governance, Jamia Millia Islamia, New Delhi, India; Santa Fe Institute, UNITED STATES

## Abstract

Dengue fever is the most important arboviral disease in the tropical and sub-tropical countries of the world. Delhi, the metropolitan capital state of India, has reported many dengue outbreaks, with the last outbreak occurring in 2013. We have recently reported predominance of dengue virus serotype 2 during 2011–2014 in Delhi. In the present study, we report molecular characterization and evolutionary analysis of dengue serotype 2 viruses which were detected in 2011–2014 in Delhi. Envelope genes of 42 DENV-2 strains were sequenced in the study. All DENV-2 strains grouped within the Cosmopolitan genotype and further clustered into three lineages; Lineage I, II and III. Lineage III replaced lineage I during dengue fever outbreak of 2013. Further, a novel mutation Thr404Ile was detected in the stem region of the envelope protein of a single DENV-2 strain in 2014. Nucleotide substitution rate and time to the most recent common ancestor were determined by molecular clock analysis using Bayesian methods. A change in effective population size of Indian DENV-2 viruses was investigated through Bayesian skyline plot. The study will be a vital road map for investigation of epidemiology and evolutionary pattern of dengue viruses in India.

## Author Summary

Dengue is a mosquito borne disease prevalent in more than 100 tropical and sub-tropical countries. In this study, we carried out phylogenetic and molecular clock analysis of dengue serotype 2 strains found to be circulating in Delhi, India, in 2011–2014. All the study strains were found to belong to the Cosmopolitan genotype of dengue 2 viruses. Further we also detected a lineage replacement event in the 2013 dengue fever outbreak. We identified a novel mutation in the stem region of the Envelope protein. We also determined nucleotide substitution rate and time to the most recent common ancestor of dengue 2 viruses. Further a change in the population size of dengue 2 viruses in India as depicted by Bayesian Skyline plot is reported.

## Introduction

Dengue fever (DF) is the most prevalent arthropod-borne disease in the tropical and sub-tropical regions of the world. It is estimated that 2.5 billion people are at risk of dengue infection globally, 50 million dengue virus infections occur annually and 500,000 people with Dengue haemorragic fever (DHF) require hospitalization every year [1]. Dengue virus (Family Flaviviridae, Genus Flavivirus) is a small enveloped RNA virus carrying a single stranded, positive-sense RNA genome (~10.6 kb). The genome encodes three structural and seven non-structural proteins in a single open reading frame. The structural proteins are capsid (C), precursor membrane (prM), and envelope (E) proteins. The envelope glycoprotein is essential for viral entry into the cell. Non-structural proteins are involved in viral replication within the cell, named NS1, NS2A, NS2B, NS3, NS4A, NS4B, and NS5.

Dengue virus (DENV) is transmitted primarily by *Aedes aegypti* mosquitoes. Common symptoms of DF are fever, fatigue, rash, headache, retro-ocular pain, arthralgia, myalgia, nausea, vomiting and low platelet count. While most infections result in asymptomatic response or mild febrile illness (DF), a small percentage of cases result in the more severe and potentially fatal dengue with warning signs and severe dengue which are characterized by plasma leakage [2,3]. There are four antigenically distinct, closely related serotypes of the dengue virus (DENV1–4), exhibiting a 65–70% sequence homology [2]. A vaccine for dengue is not available yet because of its 4 serotypes which enhance the risk of severe disease by antibody dependent enhancement of infection [4].

Dengue fever outbreaks occur after every 3–4 years in Delhi, India. Recently, dengue fever outbreaks were reported in Delhi in 2003 [5], 2006[6], 2010 [7] and 2013[8]. We have studied dengue prevalence and serotypic distribution in the period 2011–2014 in Delhi [9]. We detected three dengue serotypes (DENV-1, 2 and 3) in circulation and predominance of DENV-2 in Delhi in the above mentioned study. The present study focuses on the molecular characterization and evolutionary analysis of only DENV-2 strains detected in the study. The same analysis for DENV-1 and 3 has been published elsewhere [9].

## Materials and Methods

### Ethics statement

The study was granted approval by Institutional Ethics Committee, Jamia Millia Islamia and was done in accordance with the World Medical Association Declaration of Helsinki. Written informed consent in English or Hindi was obtained from all adult subjects or a parent or guardian in case of minors.

### RT-PCR and sequencing

Acute phase blood samples were collected from suspected dengue patients from Dr. M. A. Ansari Health Centre, Jamia Millia Islamia, New Delhi. Sera were separated from the blood samples by centrifuging at 3000 rpm for 10 minutes at 4°C. Serum samples were stored at -80°C until further use. RNA was extracted from 140 μL serum samples using QIAamp Viral RNA Mini kit (Qiagen, Hilden, Germany) according to the manufacturer’s instructions. cDNA synthesis was carried out in a 25 μL reverse transcription (RT) reaction mixture using 20 ng of random primers (Promega, USA), 1mM dNTPs (Promega, USA), 8U of rRNAsin (Promega, USA), 10U of Avian Myeloblastosis Virus reverse transcriptase (Bangalore Genei, India) and 13.75 μL RNA. Detection of dengue viruses were carried out by semi nested RT-PCR method as reported previously [10] with some modifications [8,11].

The E gene (574bp) of DENV-2 viruses was amplified using another set of published primers for DNA sequencing [12]. Amplicons were run on agarose gel and the bands were cut. Gel extraction was done using QIAquick Gel Extraction Kit (Qiagen, Germany) as per the manufacturer’s instructions. Sequencing with both forward and reverse primers was done commercially (Xcelris Labs, Ahmedabad, India).

### Phylogenetic analysis using MEGA

The raw sequences were subjected to similarity search using BLAST. Forward and reverse sequences were aligned and manually edited with GeneDoc (v2.7.000) software. Sequences were aligned with other published sequences available in GenBank using Clustal W implemented in BioEdit (v7.0.9.0). The best fit substitution model for the data was determined by Akaike Information Criterion (AIC) using MODELTEST 3.7 [13]. Phylogenetic tree was constructed using Maximum likelihood method in Mega 6.06 software [14]. Statistical support for the nodes was assessed by bootstrapping with 1000 replicates.

### Bayesian MCMC molecular dating analysis

Rate of nucleotide substitution and Time to the most recent common ancestor (TMRCA) of DENV-2 strains were assessed using Bayesian inferences implemented in BEASTv1.8.1 [15]. TN93+G+I model was used as the nucleotide substitution model and among site rate variation model (selected by MODELTEST3.7). Both strict and relaxed (uncorrelated exponential and uncorrelated lognormal) molecular clocks [16] were used for the analysis. Constant size and Bayesian skyline coalescent tree prior were used in the study. The MCMC chain was run for 30,000,000 steps. The parameter values were sampled at every 3000 steps. Each analysis was performed in two separate runs and the resulting log files were combined using LogCombiner 1.8.1 (implemented in BEAST) with 10% burn-ins removed from each run. The resulting log files were analysed in the program Tracer 1.6 to ascertain convergence of the chain and to ensure that effective sample size of >200 for all parameters have been reached. The uncertainty in the parameter estimates were assessed by 95% HPD intervals. Model comparison was done by Bayes factor (Log Marginal Likelihood (M1)-Log Marginal Likelihood (M2)). The maximum clade credibility tree was generated by Tree Annotator 1.8.1 (available in BEAST), and the resulting tree file was visualized in the program FigTree 1.4.2. Support for the node on the tree was ascertained by the Bayesian posterior probability (BPP) values for each node. The BEAST package was also used to infer Bayesian skyline plots for Indian cosmopolitan DENV-2 strains (n = 24, group size = 10). This analysis enabled a graphical depiction of changing levels of relative genetic diversity (*Ne*τ, where *Ne* is the effective population size and τ the host-to-host generation time) through time.

### Selection pressure analysis

The selection pressure acting on envelope gene codons of DENV-2 was investigated using the online facility at the web server http://www.datamonkey.org. Two datasets were used for this analysis, one dataset comprising strains from all DENV-2 genotypes (n = 58) and second dataset comprising of only the Cosmopolitan genotype (n = 30) strains. TN 93 model of nucleotide substitution and Neighbour Joining tree was used for the analysis. The ω ratios (dN/dS) were calculated using three likelihood approaches, single-likelihood ancestor counting (SLAC), fixed effects likelihood (FEL) & Random effects likelihood (REL). Sites showing evidence of positive selection by at least two of the methods with high statistical significance (P < 0.1 or Bayes factor >50), were considered to be under positive selection.

### T-cell and B- cell epitope prediction

T-cell epitopes of the envelope gene of prototype dengue 2 virus were predicted using the EpiJen online server [17]. Envelope protein sequence of DENV-2 prototype strain (NGC 44 strain; GenBank Accession Number: AF038403) was submitted for epitope prediction. Appropriate proteasomal and Tap cut-off values were selected and epitopes were predicted for 18 different HLA alleles. B cell epitopes were predicted using the BCPreds prediction tool [18]. The predicted B-cell epitopes were further analyzed by VaxiJen server (antigenicity prediction server based on auto cross variance (ACC) transformation of protein sequences into uniform vectors of principal amino acid properties). The B cell epitope having BCPreds score of >0.8 and VaxiJen score of >0.6 were selected as the predicted B- cell epitope.

### Mapping of mutations

The structure for the partial envelope gene sequence (152 amino acid) obtained in the study was modeled using I-TASSER protein structure prediction tool. The structure was visualized in Pymol and amino acid mutations (E322 and E404) were mapped on the structure using Pymol.

### Statistical analysis

The significant number of samples to be sequenced for each lineage (I and III) was calculated by taking into account the total number of samples year wise with the Epi-Info software (http://wwwn.cdc.gov/epiinfo/) using the epidemiologic calculator (StatCalc). The statistical calculation of the Population Survey was done using the Sample size and power option in the Epi-Info software using number of samples collected during the study period (for the years 2012, 2013 and 2014) and the expected frequency of Dengue virus infection at 5% level of significance. In addition, the analysis of lineage replacement with respect to total cases taken year wise was done with chi-square test. A p-value less than 0.05 was considered significant.

## Results

**Fig 1:**
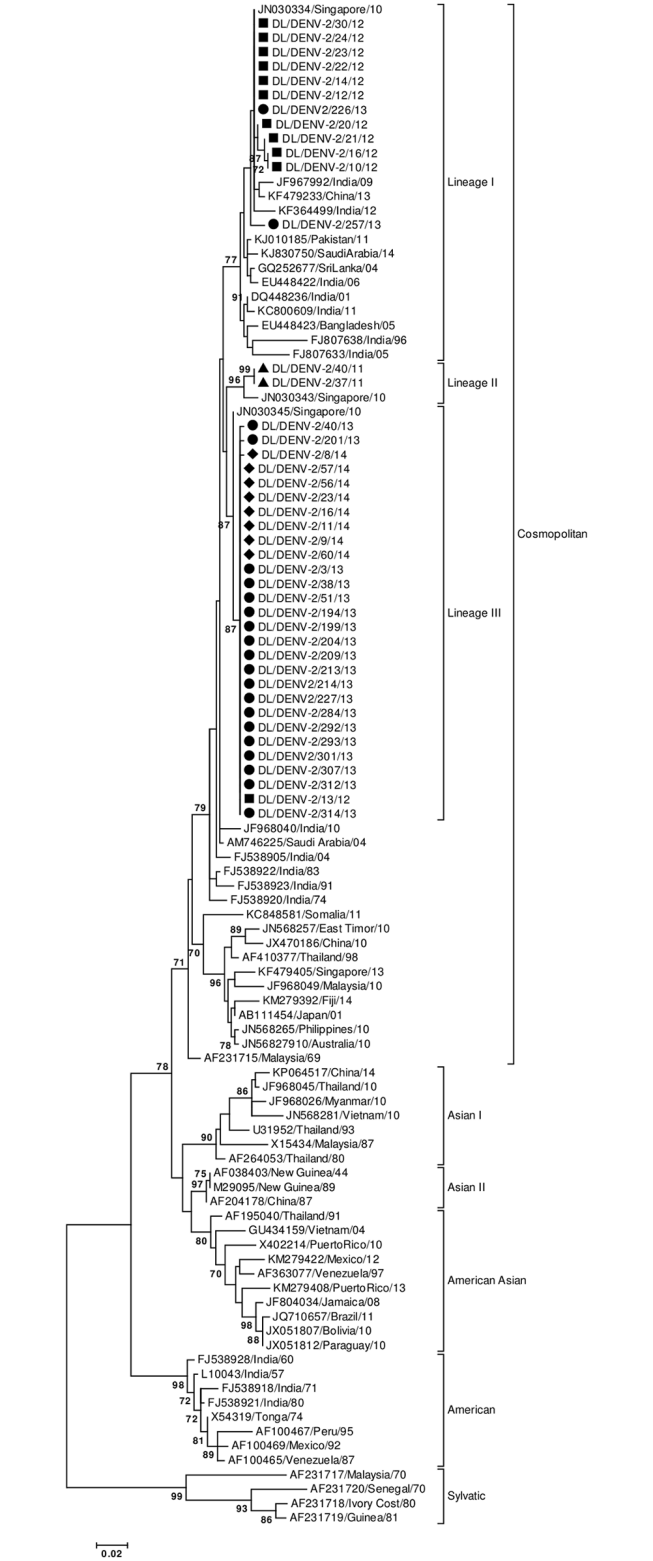
Maximum Likelihood Phylogenetic tree of DENV-2 strains. Strains sequenced in the study are marked by shapes (2014 strains: diamonds; 2013 strains: circles; 2012 strains: rectangles; 2014 strains: triangles). Numbers on nodes indicate bootstrap support generated by 1000 replicates. Bootstrap values of >70 are shown.

Envelope gene of 42 DENV-2 strains was sequenced in the study (GenBank Accession Numbers: KJ729151-KJ729162, KM875638- KM875659; KR091039-KR091046). Sequencing and phylogenetic analysis of sequences with accession numbers KJ729151-KJ729162 have been described in our previous publication [8]. Eight DENV-2 strains were sequenced in 2014, 21strains in 2013, 11 strains in 2012 and 2 strains in 2011. A total of 106 sequences were used in the phylogenetic analysis with 58 Indian strains. The 457bp (152aa) long multiple sequence alignment corresponded to 1801–2257 bp of the DENV-2 genome and 289–440 amino acid of the E protein (numbering based on the prototype NGC 44 strain; GenBank Accession Number: AF038403). Maximum Likelihood phylogenetic tree of the strains based upon the aligned sequence data showed that the study strains clustered with the Cosmopolitan genotype (Fig 1). Strains from Pakistan, China, Singapore, Bangladesh, Sri Lanka and Saudi Arabia were found to cluster with the strains of the present study. DENV-2 strains of the study clustered in three different lineages i.e. Lineages I, II and III (Fig 1). Strains of 2014 clustered in lineage III, those detected in 2013 and 2012 clustered in lineage I and III while strains of 2011 clustered in lineage II (Fig 1). A single amino acid change, I322V, was detected between the strains of 2011–2014 from Delhi. Lineage I strains (n = 12) showed Valine at position 322 while Lineage II (n = 2) and III (n = 28) strains showed Isoleucine at this position. The amino acid mutation at 322 position was mapped to two T-cell epitopes and one B- cell epitope (Table 1). When year wise prevalence of lineage I and III strains was analyzed, a pattern of alternation in predominance of the lineages I and III in 2012–13 was detected. Lineage I strains were found in 10 samples while lineage III strain in only one sample (ratio of 10:1) in 2012 showing predominance of lineage I over lineage III strain. In 2013, Lineage I was detected in only 2 samples while Lineage III strains were detected in 19 samples (ratio of 1:10). Thus a transition to complete lineage replacement was identified in 2013. All 2014 study strains belonged to Lineage III thus further giving support to the lineage replacement event.

**Table 1:** Correlation of mutations in the envelope protein of the study strains with the predicted T and B cell epitopes.

Mutation	Position	T-cell/ B cell epitope	Epitope	HLA alleles
I322V	314	T- cell epitope	ETQHGTIV**I**	HLA-A*6802
I322V	318	T- cell epitope	GTIV**I**RVQY	HLA-A*0101
I322V	317	B- cell epitope	HGTIV**I**RVQYEGDGSPCKIP	-
T404I	401	T- cell epitope	MIE**T**TMRGA	HLA-A*0301, HLA-A*1101
T404I	402	T- cell epitope	IE**T**TMRGAK	HLA-B *40, HLA-B*44
T404I	403	T- cell epitope	E**T**TMRGAKR	HLA-A*0101, HLA-A*0301, HLA-A*3101

Position of the epitope is with respect to the E protein of Dengue virus 2 prototype strain (NGC 44)

Statistical analysis for giving credence to the lineage replacement was also carried out. The significant number of samples to be sequenced was calculated using the following parameters: total number of samples were 77, 378 and 62 for the year 2012, 2013 and 2014 respectively, the expected frequency of Dengue virus infection was 50± 25%. At the 5% level of significance, the number of samples that should be sequenced was calculated to be 13, 15 and 12 for the year 2012, 2013 and 2014 respectively. Our analysis showed that significant number of samples were sequenced for each lineage during the study. Further statistical analysis concluded that replacement of lineage I by lineage III is independent of the total number of samples taken year wise (p = 0.0001 for lineage I and p = 0.009 for lineage III).

Lineage I comprised of Indian strains detected between the years 1996 and 2012. Lineage I was also found as a major lineage in 2012. Lineages II and III included strains found in India in the period 2011–2014. Apart from Delhi strains, the Lineage II included a solitary strain from Singapore. Lineage III was detected for the first time in 2012 and continued till 2014 in Delhi. It was a minor lineage in 2012 but gained predominance in 2013 replacing lineage I. No other previously reported Indian strains (except the study strains) clustered within the newly emerged Lineage II and III.

**Fig 2:**
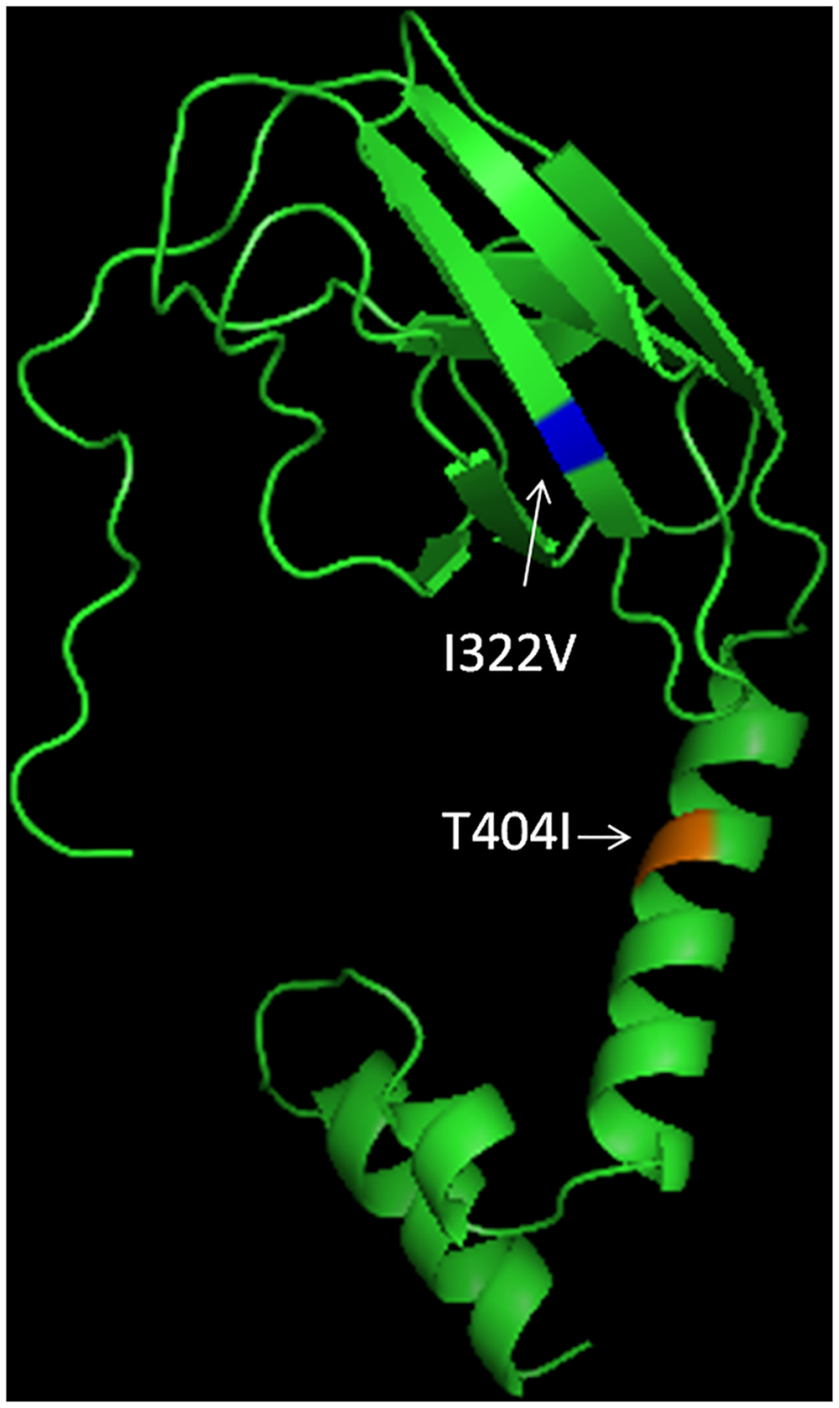
Modelled structure of Envelope Protein (289–440) showing position of I322V & T404I mutations.

The study strains showed nucleotide distance of up to 5.4% whereas amino acid distances were up to 1.3%. Nucleotide distance of 6.4 to 7.2% and amino acid distance of 1.3–2% were detected when compared to the prototype strain. Two mutations, Asn390Ser and Ile402Phe in comparison to the prototype strain were detected in all study strains which are both previously reported [19]. A novel mutation Thr404Ile (S1 Fig) was detected in a single strain (DL/DENV-2/8/14) in 2014 highlighting the importance of molecular epidemiology studies in revealing such mutations. The mutation was confirmed by sequencing twice with forward and reverse primers. This mutation was mapped to three T-cell epitopes predicted by Epi-Jen server (Table 1). The deduced amino acid sequence of the strain DL/DENV-2/8/14 was used to predict its structure. Amino acid positions 322 and 404 were mapped on the modelled structure (Fig 2).

### Bayesian MCMC analysis

**Fig 3:**
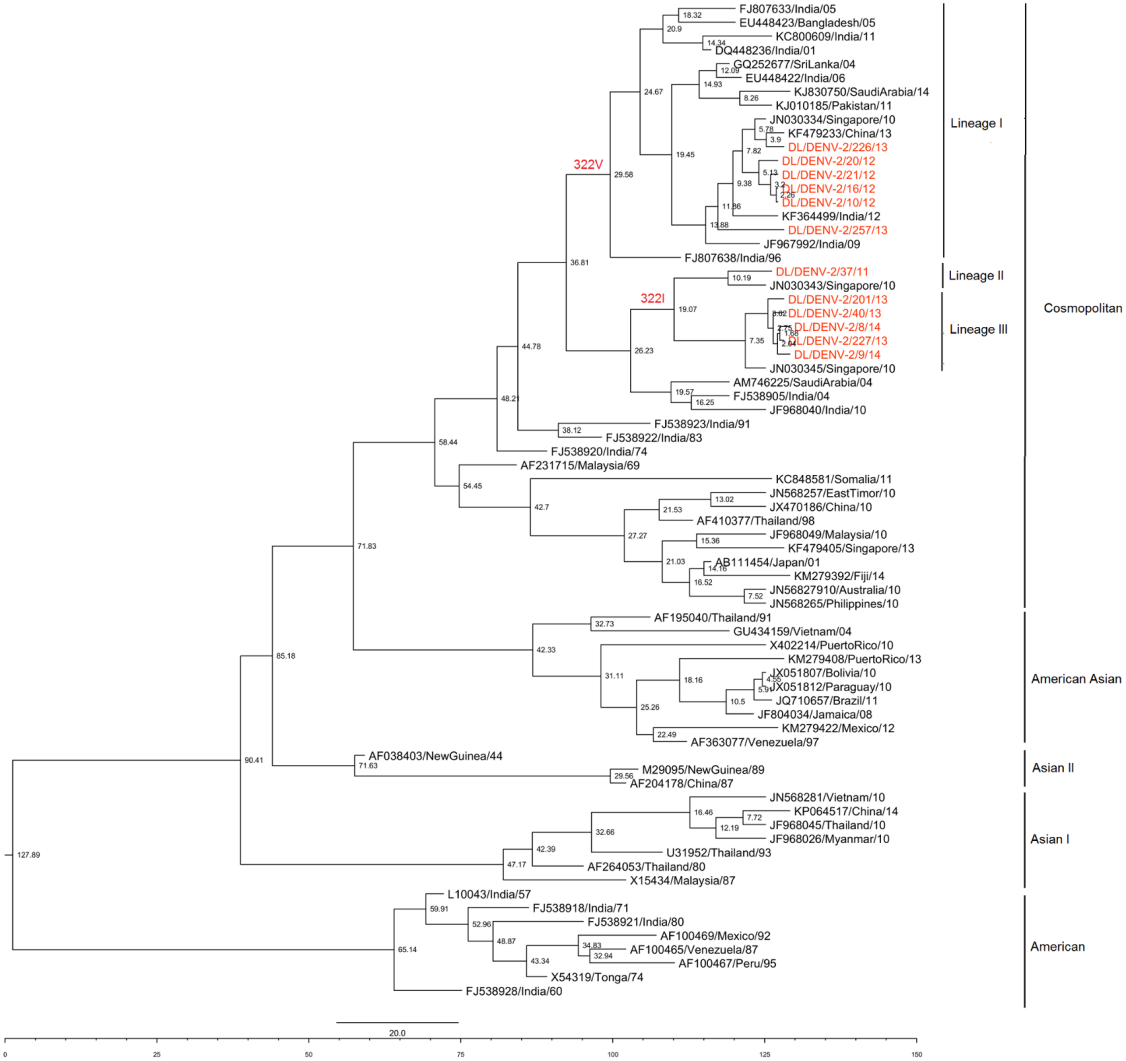
Maximum Clade Credibility tree of Dengue 2 virus. Tree derived with the best fit model (relaxed uncorrelated lognormal clock & Bayesian skyline tree prior) showing node ages. Strains sequenced in the present study are coloured. Lineages showing Valine or Isoleucine at position 322 have been marked.

Uncorrelated relaxed lognormal clock and Bayesian coalescent tree prior was chosen as the best fit model as they were favoured by Bayes factor (S1 Table). Nucleotide substitution rate was detected to be 7.7 ×10^−4^ substitutions per site per year (95% HPD; 5×10^−4^ to 1.052 ×10^−3^) under the best fit model. Root of the tree was calculated to be 128 years old [86–194 years 95% HPD, 1886 (1820–1928)]. TMRCA of the Cosmopolitan genotype was determined to be 59 years [45–81 years 95% HPD, 1955 (1933–1969)]. TMRCA of the Indian Cosmopolitan genotype strains was determined to be 48 years [41–58 years 95% HPD; 1966 (1956–1973)]. The Maximum Clade Credibility Tree was constructed with the best model as shown in Fig 3. The tree showed three lineages of dengue 2 viruses in the period 2011 to 2014. TMRCA of Lineage I was estimated to be 30 years (1984), that of lineage II as 10 years (2004) and that of lineage III as 7 years (2007).

**Fig 4:**
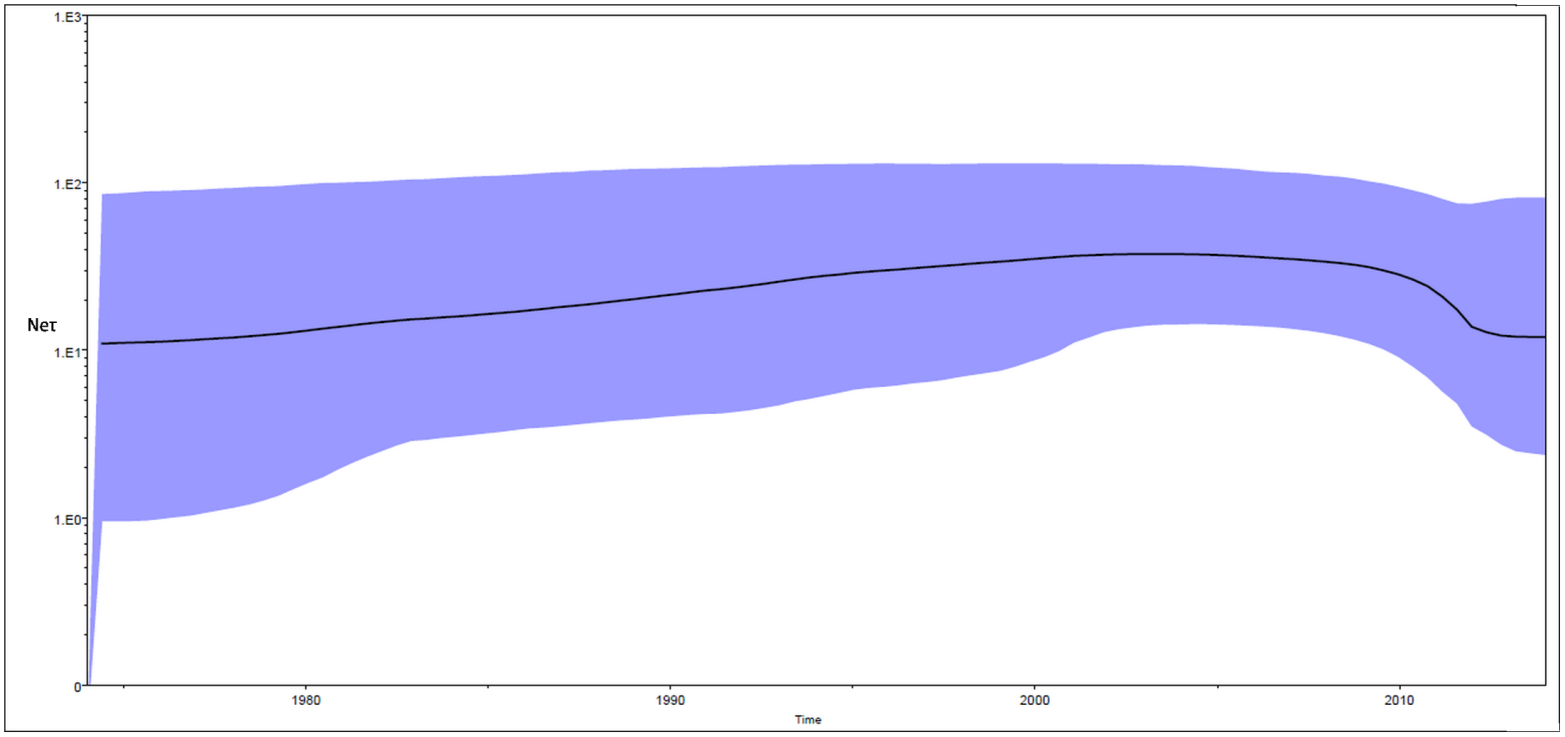
Bayesian skyline plots of Indian Cosmopolitan DENV-2 strains. X axis: Time, Y axis: Relative genetic diversity (Neτ). Black solid line is the median estimate of Neτ. Blue shaded area shows 95% HPD.

Bayesian skyline plot (BSP) for Indian Cosmopolitan strains was constructed in the study (Fig 4). The plot shows changes in the median estimate of relative genetic diversity (*Ne* τ) of the virus with time where *Ne*τ is the product of effective population size (*Ne*) and generation time (τ). The plot also shows 95% highest probability density intervals which represents both phylogenetic and coalescent uncertainty. Population size of Indian Cosmopolitan strains increased smoothly from 1974 to 2004 with a small growth rate (Fig 4). Population size remained almost constant in the period 2005 to 2009. It decreased between 2009 & 2012 and then remained constant between 2012–2014. Levels of Neτ in 2012–2014 were lower than in the start of the plot.

### Selection pressure analysis

The mean dN/dS ratio was found to be 0.05 in the codons of the E gene. There was no codon detected under positive pressure by the SLAC and FEL methods for the datasets. In the dataset comprising of all genotype strains (N = 58), three codons were found to be under positive selection pressure by the REL method i.e. E317, E337 & E365. The E322 site showing Valine in Lineage I and Isoleucine in Lineage III was detected as negatively selected by SLAC & REL method and as neutrally evolving site by FEL method in the dataset containing strains from all the genotypes. Site E404 was shown to be neutrally evolving by SLAC &FEL methods and as negatively selected by the REL method.

In the second dataset which included strains of only the Cosmopolitan genotype strains (n = 30), no codon was detected to be under positive selection pressure by SLAC, FEL & REL method. Site 322 & 404 were detected to be neutrally evolving by the SLAC & FEL methods. Site 322 was detected to be negatively selected and site 404 as neutrally evolving by the REL method.

## Discussion

Delhi is a metropolitan city of India which battles dengue outbreaks after every 3–4 years. Recently we have reported a change in dengue serotype predominance in Delhi [9]. Dengue serotype 3 predominated in 2003–2006, dengue 1 in 2008 and 2010 and dengue 2 in 2011–2014 in Delhi [9]. Dengue virus type 2 (DENV-2) has been responsible for the 1996 [20] and 2013 [8] dengue fever outbreaks in Delhi, India. In the present study DENV-2 strains detected in Delhi during 2011–2014 were analyzed phylogenetically.

All the DENV-2 strains sequenced in the study clustered in the Cosmopolitan genotype. This genotype has been reported from India since 1974 [19,21]. Earlier Indian study based upon E-NS1 gene sequencing of Delhi strains detected during 1957, 1967 and 1996 [22] reported that before the Cosmopolitan genotype, American genotype was found to be in circulation in India. This was later confirmed by CprM gene sequencing of the same strains [23]. Kumar and colleagues [19] reported that Indian strains from 1956 to 1980 belonged to the American genotype and strains from 1974 onwards clustered in the Cosmopolitan genotype. Dash and co-workers [24] have also reported change in circulating genotype in India based upon complete genomic sequencing.

Lineage replacement in dengue viruses has been reported in many studies [25–33] including India. A lineage replacement event where lineage III replaced lineage I was detected in 2012–2014 in Delhi. The statistical analysis revealed that the lineage replacement event was independent of the total number of samples analyzed year wise. We detected lineage replacement in 2013 in which a dengue outbreak occurred in Delhi [8]. It has been suggested that lineage replacement could result in increased transmission of the viral infection [32]. Thus, the lineage replacement event described in the present investigation could be related to the outbreak situation in Delhi in 2013. Lineage replacement has been also postulated to result in improved viral fitness & increased transmissibility [31,32] or as a stochastic event due to virus population bottleneck effects [30]. Lineage I strains showed Valine (V) at E322 and Lineage III showed Isoleucine (I) at E322. DENV-1 strains and DENV-4 strains have Valine at position 322 while DENV-3 strains have Isoleucine. Difference of V/I between strains might be helpful in escape of DENV-2 strains from cross neutralizing antibodies generated by DENV-1, 3 and 4 strains which target E322 [34]. Besides this difference at E322, the concerned lineages might also differ at other significant positions in the genomic regions not sequenced in the present study.

A novel mutation Thr404Ile was also detected in the study. This residue is part of the stem region (helix I) of the E protein which is essential for the formation of E protein trimers in response to low pH [35]. Site 404 is variable amongst flaviviruses showing Lysine in Tick-Borne Encephalitis Virus, Alanine in DENV-1 and 3 and Serine in DENV-4 [36]. In the strain DL/DENV-2/8/14 showing this mutation, Threonine a polar amino acid was replaced by Isoleucine which is non polar but retains the property of having a chiral carbon in the side chain. This change in the type of amino acid may have impact on the structure of the E protein. Role of this mutation in the virus life cycle is not clear at present and should be explored in the site directed mutagenesis studies.

The mutations at E322 and E404 were found in domain III and stem region respectively. These mutations may alter local secondary structure of the E protein which might affect the structure-function relationship of the protein. The mutation at E322 might influence attachment and fusion properties of the virus as domain III is the putative receptor binding domain of the E protein [37]. Mutation at E404 may influence the low pH induced fusion process of the E protein. Further these 2 sites were also mapped to predicted T-cell and B- cell epitopes. As a consequence the mutations I322V and T404I may influence immunogenicity of the dengue virus.

Nucleotide substitution rate of DENV-2 viruses calculated in the study (7.7 ×10^−4^ substitutions per site per year) is comparable to that reported in the earlier studies on complete E gene [38,39] and complete genome [40]. Kumar and colleagues [19] reported nucleotide substitution rate of DENV-2 viruses as 6.5×10^−4^ (4.1–8.7 ×10^4^, 95%HPD) substitution per site per year. Our estimate is within the 95% HPD interval reported by them. Estimates for the TMRCAs of DENV-2 strains, Cosmopolitan genotype strains and Indian Cosmopolitan strains (128, 58 & 48 years) differ from those reported in the earlier Indian study [19] but they are within the 95% HPD intervals reported in the study and are supported by smaller 95% HPD intervals. Lineage I was estimated to have TMRCA of 30 years and Lineage III showed TMRCA of 7 years. The newly emerged lineage III replaced lineage I in 2013 and 2014. Bayesian Skyline Plot of Indian Cosmopolitan DENV-2 strains have not been previously reported in literature. We show a decrease in effective population size of DENV-2 in recent years.

Thus in the present study sequencing and phylogenetic analysis of DENV-2 strains revealed that the circulating strains belonged to the Cosmopolitan genotype. A lineage replacement event and a novel mutation were also detected. Nucleotide substitution rate and TMRCA of DENV-2 viruses are also reported. Past population dynamics as studied by Bayesian skyline plots showed a decrease in the population size of DENV-2 strains in recent years. The present investigation will assist in design of effective vaccine and antivirals for control of the virus. Further, it will also help us to determine the epidemiological and evolutionary pattern of Dengue outbreaks in India.

## Supporting Information

S1 Fig Raw sequence data of DENV-2 strain DL/DENV-2/8/14 showing amino acid mutation of Threonine to Isoleucine. A. Sequence with forward primer. The sequence corresponds to position 1196–1226 of Envelope gene. The amino acid T at position 340 (1211 of Envelope gene) results in change from Threonine to Isoleucine in the deduced amino acid sequence. B. Sequence with reverse primer. Sequence complementary to 1226–1196 of Envelope gene. A at 124 (1211 of Envelope gene) results in change from Threonine to Isoleucine in the deduced amino acid sequence.
(TIF)

S1 Table Log marginal likelihoods of various models by different methods. (DOCX)
