# Supplementary figures and images for: Evolutionary Analysis of Dengue Serotype 2 Viruses Using Phylogenetic and Bayesian Methods from New Delhi, India
Source: PLoS Negl Trop Dis. 2016 Mar 15;10(3):e0004511. doi: 10.1371/journal.pntd.0004511 (PMC4792444; doi:10.1371/journal.pntd.0004511)

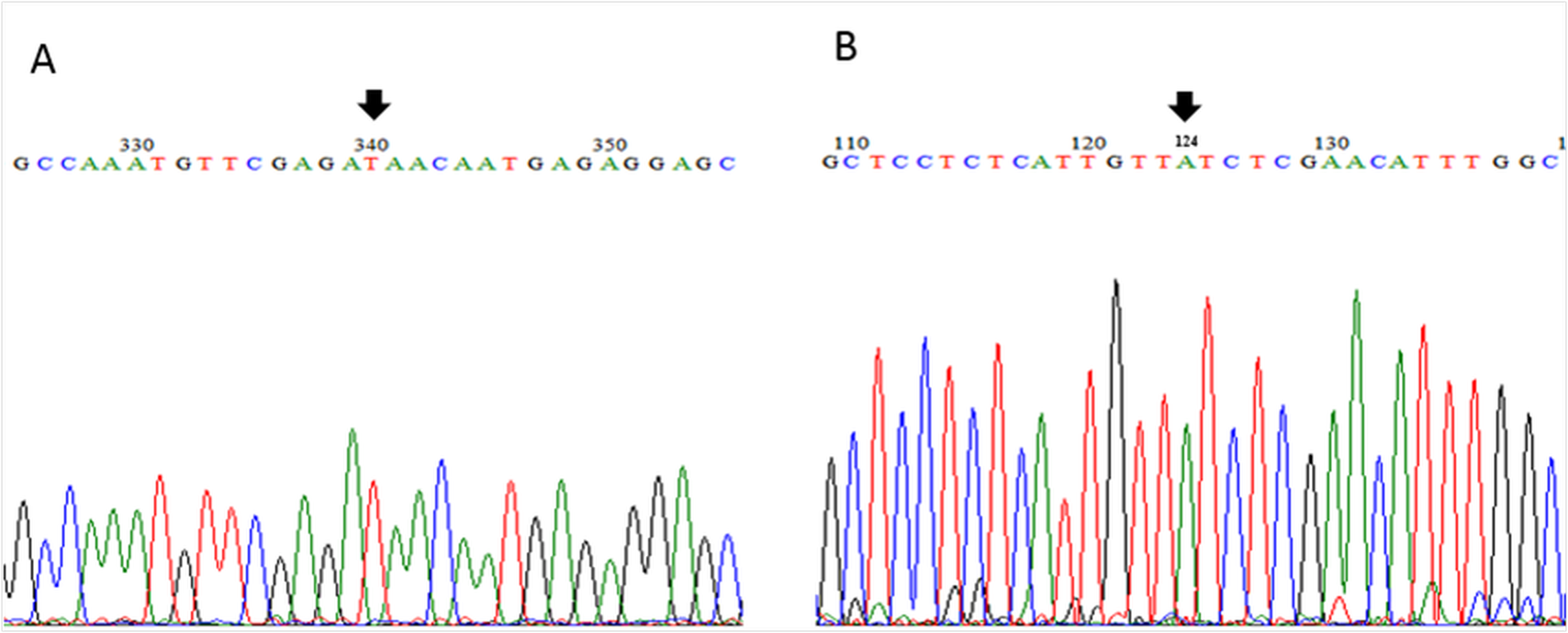

Supplement: S1 Fig — A. Sequence with forward primer. The sequence corresponds to position 1196–1226 of Envelope gene. The amino acid T at position 340 (1211 of Envelope gene) results in change from Threonine to Isoleucine in the deduced amino acid sequence. B. Sequence with reverse primer. Sequence complementary to 1226–1196 of Envelope gene. A at 124 (1211 of Envelope gene) results in change from Threonine to Isoleucine in the deduced amino acid sequence. (TIF) [file pntd.0004511.s001.tif]
