# Supplementary material for: Evolutionary Analysis of Dengue Serotype 2 Viruses Using Phylogenetic and Bayesian Methods from New Delhi, India
Source: PLoS Negl Trop Dis. 2016 Mar 15;10(3):e0004511. doi: 10.1371/journal.pntd.0004511 (PMC4792444; doi:10.1371/journal.pntd.0004511)
Supplement: S1 Table — (DOCX) [file pntd.0004511.s002.docx]

**S1 Table. Log marginal likelihoods of various models by different methods**

| **Model** | **Log Marginal Likelihood Harmonic Mean Estimator** | **AICM** | **Log Marginal Likelihood(using Path Sampling)** | **Log Marginal Likelihood (using Stepping Stone Sampling)** |
| --- | --- | --- | --- | --- |
| Strict Clock, Constant Population | -3005.44 | 6095.53 | -3282.51 | -3282.98 |
| Strict clock, Skyline | -3003.62 | 6087.55 | -3275.32 | -3275.56 |
| Uncorrelated Relaxed Lognormal, Constant Population | -2974.24 | 6084.07 | -3265.69 | -3265.61 |
| Uncorrelated Relaxed Lognormal, Skyline | -2974.77 | 6085.68 | -3263.50 | -3264.49 |
| Uncorrelated Relaxed Exponential, Constant Population | -2963.91 | 6060.60 | -3267.35 | -3267.80 |
| Uncorrelated Relaxed Exponential, skyline | -2962.69 | 6063.70 | -3266.92 | -3267.11 |
